# Extract of Conium in Inflammation of the Breast

**Published:** 1871-12

**Authors:** 


EXTRACT OF CONIUM IN INFLAMMATION OF THE BREAST. —M. Alstadter, of Pesth, strongly recommends small doses of extract of conium, repeated several times in the course of the day, for the resolution of inflammation of the breast, arising from stasis of the milk in puerperal women, and reports several cases in which striking advantage was obtained from its use. In all instances care should be taken to obtain as pure and active a specimen of the drug as possible.—*Wiener Medical Presse.*

